# Whole-genome sequencing and *de novo* assembly of *Alternaria alternata* ATCC 66981

**DOI:** 10.1128/mra.00167-25

**Published:** 2025-10-08

**Authors:** Adetoye W. Adeyemo, Markus Schmidt-Heydt

**Affiliations:** 1 Department of Safety and Quality of Fruit and Vegetables, Max Rubner-Institut, Federal Research Institute of Nutrition and Food 14878 , Karlsruhe, Germany; University of California Riverside, Riverside, California, USA

**Keywords:** *Alternaria alternata*, mycotoxins, whole-genome sequencing

## Abstract

*Alternaria alternata* is known to be a major producer of *Alternaria* toxins such as alternariol or altertoxin-I. Here, we report the genome sequencing of *A. alternata* strain ATCC 66981 on the Illumina MiSeq platform.

## ANNOUNCEMENT

**Fig 1:**
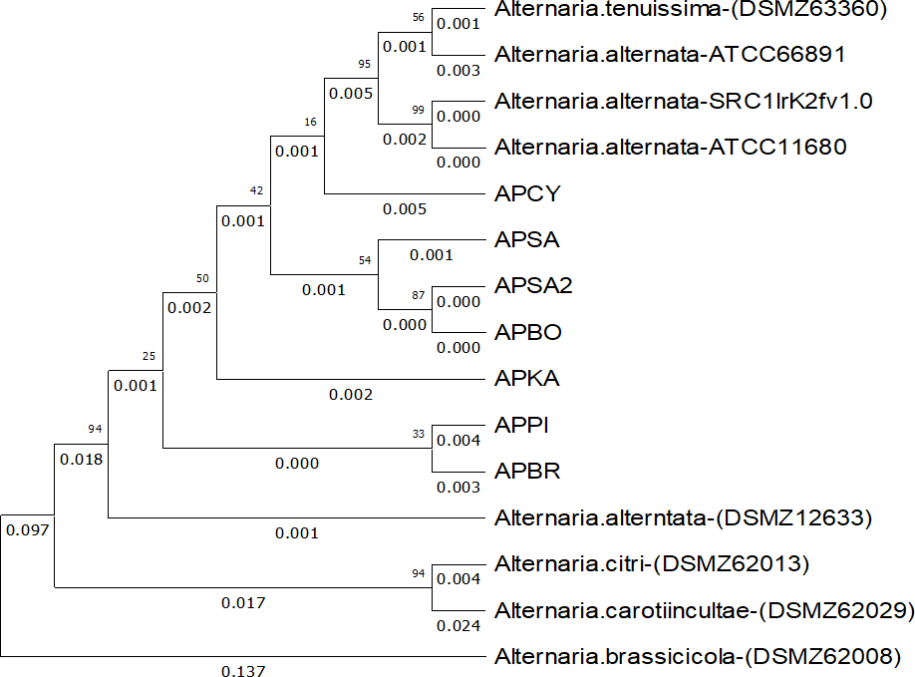
Phylogenetic tree of different *Alternaria* species. This phylogenetic tree resulted from the alignment and subsequent concatenation of the molecular markers ITS, GDP, RPB2, EndoPG, and Alta-1 by the program MEGA11 with the alignment algorithm ClustalW. The build tree is a maximum likelihood tree with 100 bootstrap replications using the general time reversible method and gamma distributed rates. In addition to ATCC66981, all strains used by Adeyemo et al. 2024 (11) were used in this phylogenetic tree as well.

The filamentous fungus *Alternaria alternata* is an important toxigenic fungus that occurs on a cosmopolitan level (1). It also has great significance as a plant pathogen due to its ability to infect a wide variety of different hosts which range from fruits to grains and even grass (2, 3). Especially, strain ATCC66981 was repeatedly used in research (4, 5) (6–9) (10). Hence, we decided to perform a *de novo* assembly with the short reads resulting from the Illumina MiSeq sequencing. Genomic DNA of *A. alternata* ATCC66981 which was drawn from our −80°C long time storage, was extracted from a pure culture grown on cellophane on top of YES agar for 10 days at 25°C in the absence of light. After scraping the mycelium from the plate and using a bead beater (FastPrep) to disrupt the sample. Then, the NucleoSpin plant II kit (Macherey-Nagel) was used to extract gDNA, which was afterwards quantified and quality checked using a NanoDrop 1000 instrument (260/280: 1.9, 260/230: 2.1) (VWR International) and Qubit 3.0 photometer (10 ng/µl), respectively. Genome sequencing was carried out on the MiSeq platform (Illumina) as follows: the sequencing library was built using the Illumina Nextera DNA XT kit and quality checked using Experion DNA 1k analysis (Bio-Rad Laboratories). *De novo* assembly was carried out with SeqMan NGen Version 17.2 DNASTAR, Inc. Madison, WI. To ensure the necessary quality, except otherwise noted, the default parameters were used; sequencing adapters, PhiX control, contigs of <200 nucleotides (nt), and mitochondrial sequences were removed. The assembly size was 33.00 Mb with 56× coverage, which contained 257 genomic scaffolds/contigs; the *N*_50_ value was 393 kb and the GC content was 51.5%. The completeness of the sequencing was verified using a BUSCO analysis resulting in a total of 1,63453 complete single-copy and 2 complete duplicated orthologs, 15 fragmented orthologs, and 46 missing orthologs . Thereafter, we verified the identification of ATCC by the alignment and concatenation of multiple molecular markers such as ITS (internal transcribed spacer), GDP (glycerol-3-phosphate dehydrogenase), RPB2 (RNA polymerase II subunit B), Alta-1 (major *A. alternata* allergen), and EndoPG (endopolygalacturonase) can be seen in Fig. 1 (11).

The sequences of the markers were retrieved by a BLASTN search. Furthermore, the prediction of biosynthetic gene clusters (BGCs) was carried out with antiSMASH fungal V7.0 alpha into which the whole genome of ATCC66981 was uploaded. Here, the cluster finder algorithm for BGC border prediction with standard settings was used (12, 13). There were 29 BGCs predicted within the genome sequence of *A. alternata* ATCC 66981 distributed as follows: seven T1PKS clusters, three terpene clusters, five NRPS clusters, six fungal-RiPP-like clusters, one NAPAA cluster, and six NRPS-like clusters. Gene clusters with gene sequence similarity to known clusters are listed in Table 1. The predictions of the presence of the 1,3,6,8-tetrahydroxynaphthalene (T4HN) cluster and the alternariol biosynthesis cluster align with the findings of ATX and AOH synthesis in ATCC 66981 (7, 8).

**TABLE 1:** BGCs predicted with fungal antismash^*a*^

BGC type	Predicted BGC	Gene sequence similarity (gss)
T1PKS cluster	T4HN	100%
T1PKS cluster	Abscisic acid synthetic cluster	50%
T1PKS cluster	Alternariol biosynthesis cluster	100%
T1PKS cluster	Alternapyrone cluster	80%
Terpene cluster	Squalestatin S1	40%
NRPS cluster	Metachelin biosynthetic cluster	50%
NRPS cluster	Equisetin gene cluster	45%
NRPS cluster	Choline biosynthetic cluster	100%

^*a*^ BGC, biosynthetic gene cluster; T4HN,1,3,6,8-tetrahydroxynaphthalene.

Additionally, to the predicted BGCs, a putative *A. alternata* sulfotransferase with the accession PP982769.1 was found. Deeper analysis of the beforehand mentioned gene clusters expressed in *Alternaria* spp. will improve the understanding of the physiology of *Alternaria* mycotoxin biosynthesis.

## Data Availability

This Whole Genome Shotgun project has been deposited at DDBJ/ENA/GenBank under the accession JBLEAD000000000. The version described in this paper is version JBLEAD010000000. The Bioproject can be found under the accession PRJNA1190684 and the SRA und the accession of SRX27652631.
